# Efficacy of Teleconsultation Versus Usual Care in Improving Quality of Life Among Geriatric Patients Aged 60 and Above: A Randomized Controlled Trial

**DOI:** 10.7759/cureus.68476

**Published:** 2024-09-02

**Authors:** Prakash Mahala, Nidhi Kaeley, Vartika Saxena, Smriti Arora, Yogesh Bahurupi, Vasantha Kalyani

**Affiliations:** 1 Emergency Medicine, All India Institute of Medical Sciences, Rishikesh, Rishikesh, IND; 2 Community and Family Medicine, All India Institute of Medical Sciences, Rishikesh, Rishikesh, IND; 3 Pediatrics and Child Health, College of Nursing, All India Institute of Medical Sciences, Rishikesh, Rishikesh, IND; 4 Palliative Care, College of Nursing, All India Institute of Medical Sciences, Rishikesh, Rishikesh, IND

**Keywords:** randomized controlled trial, usual care, quality of life, geriatric population, teleconsultation

## Abstract

Introduction

The geriatric population, aged 60 years and older, is rapidly growing worldwide. This demographic shift has led to a higher prevalence of chronic diseases, comorbidities, and functional impairments, placing immense pressure on healthcare systems. Teleconsultation, which uses telecommunication technologies to deliver healthcare services remotely, offers a potential solution. This study aimed to assess the efficacy of teleconsultation versus usual care in improving the quality of life among the geriatric population aged 60 years and older.

Methods

A parallel, randomized controlled trial with a 1:1 allocation ratio was conducted. Eligible participants, aged 60 and above, were recruited at the triage emergency department (ED) following a baseline eligibility assessment. Inclusion criteria included the ability to communicate in English or Hindi, possession of International Organization for Standardization (ISO-certified) instruments for self-monitoring, and willingness to comply with study procedures and provide written consent. Participants were randomly assigned to experimental and control arms using a computer-generated sequence, with allocation concealment achieved through sequentially numbered opaque sealed envelopes (SNOSEs), which were opened in front of participants after obtaining baseline data. A total of 2,000 participants (1,000 per arm) were enrolled and randomly assigned to either the teleconsultation or usual care group.

Results

In the teleconsultation group (n=1,000), 36.5% of participants (365) were female and 63.5% of participants (635) were male. Similarly, in the usual care group (n=1,000), 37.1% of participants (371) were female and 62.9% of participants (629) were male. The teleconsultation group significantly outperformed the usual care group in several domains pre-intervention, with higher mean scores in the physical health domain (11.16 vs. 10.96, P = 0.009), psychological domain (11.74 vs. 11.62, P = 0.020), and environment domain (12.44 vs. 12.26, P = 0.0001). No significant difference was observed in the social relationships domain (P = 0.452). The teleconsultation group significantly outperformed the usual care group in all domains post-intervention, with higher mean scores in the physical health domain (14.49 vs. 12.74), psychological domain (13.75 vs. 12.35), social relationships domain (14.05 vs. 12.90), and environment domain (13.91 vs. 12.94) (P < 0.001 for all).

Conclusion

These findings suggest that teleconsultation significantly improves the quality of life for elderly patients by providing a more accessible and convenient means of healthcare delivery and addressing the physical, emotional, and social challenges associated with chronic illnesses.

## Introduction

The geriatric population, individuals aged 60 and older, represents a rapidly growing segment of the global population [1]. This demographic shift results in a higher incidence of chronic diseases, comorbidities, and functional impairments, increasing the demand for healthcare services [2]. This growing need places significant strain on healthcare systems, necessitating innovative solutions such as teleconsultation, which uses telecommunication technologies to deliver remote healthcare services [1,2].

Teleconsultation provides a personalized approach to healthcare, addressing the unique needs of elderly patients by overcoming the limitations of in-person consultations, such as time and scheduling constraints. It enables more frequent and detailed interactions between patients and healthcare providers, leading to accurate diagnoses and effective treatment plans, which, in turn, improves health outcomes [2]. Additionally, teleconsultation bridges geographical barriers, particularly benefiting those in rural and underserved areas where access to healthcare providers is limited. For elderly patients in these regions, it facilitates timely and specialist care without the burden of travel, making it easier to manage multiple chronic conditions [3].

The convenience of teleconsultation enhances patient satisfaction by reducing the time and effort required for medical appointments, leading to more frequent and timely interactions between patients and providers. This increased accessibility contributes to better health outcomes and a higher quality of life (QOL) for elderly individuals, who often face significant physical, emotional, and social challenges due to chronic illnesses and frequent hospital visits [4,5].

Despite encouraging initial findings, there is a critical need for robust randomized controlled trials (RCTs) to conclusively determine the efficacy of teleconsultation compared to traditional care in the geriatric population. Our study was designed to address this gap by evaluating the effectiveness of teleconsultation in improving the QOL among elderly individuals. Through a rigorously conducted RCT, we aimed to provide clear evidence on whether teleconsultation offers a significant advantage over usual care for this demographic.

## Materials and methods

Study design

**Figure 1:**
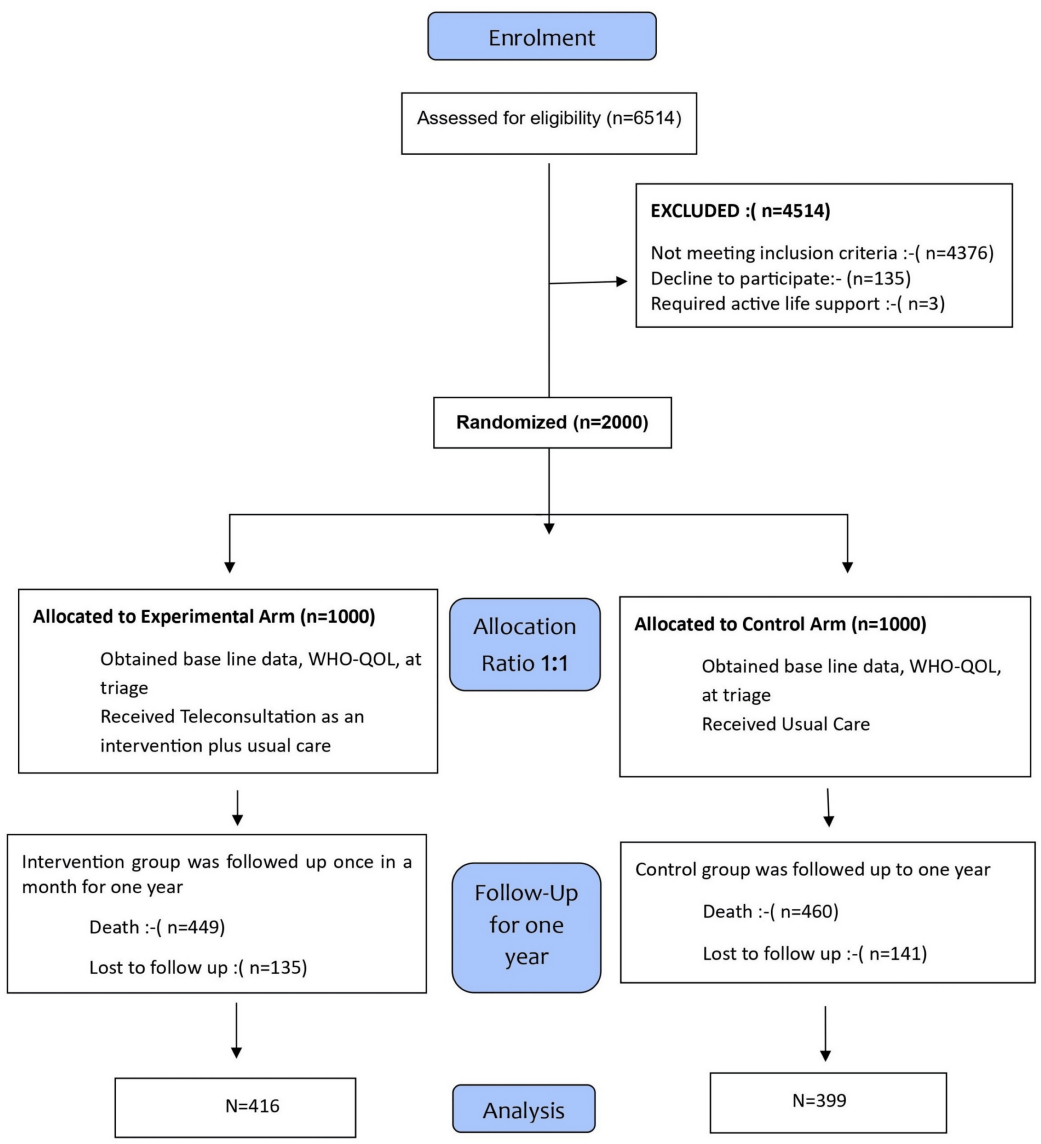
Flowchart of the study WHO-QOL, World Health Organization Quality of Life

This unstratified block RCT [6], with a 1:1 allocation ratio, was conducted at a tertiary care hospital in North India, the largest hospital in the region, serving the hilly population of Uttarakhand. The research protocol was approved by the Institute Ethics Committee of the All India Institute of Medical Sciences (AIIMS) Rishikesh, Rishikesh, India (AIIMS/IEC/21/239) and registered with the Clinical Trial Registry of India (CTRI/2021/05/033862). The study was conducted from September 2021 to December 2023. Eligible participants were contacted during their presentation at the triage emergency department (ED) and were asked for voluntary enrolment after a baseline assessment for eligibility. A total of 2,000 participants (1,000 in each arm) were enrolled after obtaining written informed consent. Baseline observations, along with the World Health Organization Quality of Life - BREF (WHO-QOL BREF) [7], were recorded on a case report form, after which participants were randomly allocated to the experimental and control arms. The inclusion criteria required participants to be over 60 years of age, able to communicate in English or Hindi, possess International Organization for Standardization (ISO-certified) instruments at home for self-monitoring, and be willing to comply with the study procedures and provide written consent. Participants were excluded if they required active life support. Participants in the experimental arm received teleconsultation as an intervention, which included usual care (with physical care if needed) and case management through the monitoring of biochemical and hematological investigations via telephone. Patients were contacted monthly for one year after discharge. Key health parameters, such as blood pressure, pulse, blood glucose level, oxygen saturation, and weight, were measured using ISO-certified home-based devices tailored to their medical needs. In contrast, participants in the control arm did not receive any specific intervention and continued with their usual care, including physical visits and care provided by emergency healthcare providers. They were also followed for one year, with outcome assessments conducted at the end of the year to compare with the experimental arm (Figure 1).

Study participants and setting of the study

The study population comprised elderly patients aged over 60 years who presented at the triage of the ED of AIIMS Rishikesh.

Randomization and blinding

Unstratified block randomization was used. Participants were allocated to the experimental and control arms using a computer-generated random sequence. Sequentially numbered opaque sealed envelopes (SNOSEs) were used for allocation concealment. All envelopes were opened in front of the participants after baseline data were obtained [6,8].

Sample size estimation

The required sample size was 915 participants per arm to detect a significant difference in the primary outcome measure with 80% power and a 0.05 significance level. A 10% attrition rate was included, bringing the total number of participants to 1,000 per arm. Sample size calculations were performed using G*Power Version 3.1.9.4 [9], with the statistical test typeset to "t-test: means, difference between two independent means (two groups)." The proportion of hospitalization in the study was used as the endpoint parameter [10].

Statistical analysis

Baseline and follow-up data were recorded on a case report form. The data were entered and stored in MS Excel 2016 (Microsoft Corp., Redmond, WA) and analyzed using SPSS Version 23.0 (IBM Corp., Armonk, NY). Descriptive statistics were used for both qualitative and quantitative variables. Independent t-tests and paired t-tests were employed for normally distributed data, while the Wilcoxon rank-sum test and Wilcoxon signed-rank test were used for non-normally distributed data. A P-value of less than 0.05 was considered statistically significant. An intention-to-treat analysis was performed to minimize attrition bias.

## Results

Table 1 shows that the gender distribution between the teleconsultation and usual care groups was similar, with no statistically significant difference (P = 0.781). The teleconsultation group had a significantly younger mean age (68.04 years) compared to the usual care group (69.70 years) (P = 0.0001). Educational qualifications showed significant differences, with a higher proportion of participants in the teleconsultation group having a graduate level or higher education (P < 0.001). There were no significant differences in family type, marital status (except for widowed participants), or religious affiliations between the groups.

**Table 1:** Comparison of demographic variables distribution between groups BMI, body mass index; SD, standard deviation

Parameters		Group				P-value
		Teleconsultation group		Usual care group		
		Frequency	Percentage	Frequency	Percentage	
Gender	Female	365	36.5%	371	37.1%	0.781
	Male	635	63.5%	629	62.9%	
Mean age (in years), mean ± SD		68.04±5.98		69.70±6.96		<0.001
Weight (in kg), mean ± SD		58.85±9.23		57.12±9.18		0.0001
Height (in cm), mean ± SD		165.87±7.63		165.39±7.92		0.162
BMI, mean ± SD		21.36±2.93		20.84±2.79		0.0001
Type of family	Joint	134	13.4%	157	15.7%	0.154
	Nuclear	866	86.6%	843	84.3%	
Educational qualification	Graduation and above	144	14.4%	58	5.8%	<0.001
	Secondary	196	19.6%	179	17.9%	
	Primary	249	24.9%	252	25.2%	
	Not at all	411	41.1%	511	51.1%	
Marital status	Divorced	0	0.0%	1	0.1%	0.675
	Married	931	93.1%	894	89.4%	
	Single	1	0.1%	2	0.2%	
	Widowed	68	6.8%	103	10.3%	
Religion	Christian	3	0.3%	0	0.0%	0.778
	Hindu	936	93.6%	896	89.6%	
	Muslim	55	5.5%	101	10.1%	
	Sikh	6	0.6%	3	0.3%	

Table 2 compares pre-intervention QOL domain scores between the teleconsultation and usual care groups. The teleconsultation group showed significantly higher scores in the physical health domain (mean 11.16, SD = 1.83 vs. 10.96, SD = 1.88; P = 0.009) and the psychological domain (mean 11.74, SD = 1.08 vs. 11.62, SD = 1.14; P = 0.020). For the social relationships domain, there was no significant difference between the groups (teleconsultation mean 11.61, SD = 2.00 vs. usual care mean 11.53, SD = 2.02). In the environment domain, the teleconsultation group had a significantly higher score (mean 12.44, SD = 1.12 vs. 12.26, SD = 1.06; P = 0.0001). These findings suggest that the teleconsultation group had better outcomes in physical health, psychological well-being, and environmental satisfaction compared to the usual care group.

**Table 2:** Comparison of baseline QOL domain between groups SD, standard deviation; QOL, quality of life

Domain	Group	N	Mean	SD	P-value
Physical health domain	Teleconsultation group	1000	11.16	1.83	0.009
	Usual care group	1000	10.96	1.88	
Psychological domain	Teleconsultation group	1000	11.74	1.07	0.020
	Usual care group	1000	11.62	1.14	
Social relationships domain	Teleconsultation group	1000	11.61	1.99	0.452
	Usual care group	1000	11.53	2.02	
Environment domain	Teleconsultation group	1000	12.44	1.12	0.0001
	Usual care group	1000	12.26	1.06	
Total QOL baseline	Teleconsultation group	1000	46.96	4.97	0.004
	Usual care group	1000	46.38	5.08	

Table 3 presents post-intervention QOL domain scores for the teleconsultation and usual care groups. The teleconsultation group achieved significantly higher scores across all domains: physical health (mean = 14.48, SD = 1.34 vs. mean = 12.73, SD = 2.33; P < 0.001), psychological (mean = 13.74, SD = 1.16 vs. mean = 12.35, SD = 1.27; P < 0.001), social relationships (mean = 14.05, SD = 1.24 vs. mean = 12.90, SD = 2.39; P < 0.001), and environment (mean = 13.90, SD = 1.00 vs. mean = 12.94, SD = 1.20; P < 0.001). These findings suggest that teleconsultation significantly improves QOL.

**Table 3:** Comparison of post-intervention QOL domain between groups SD, standard deviation; QOL, quality of life

Domain	Group	N	Mean	SD	P-value
Physical health domain	Teleconsultation group	416	14.48	1.34	<0.001
	Usual care group	399	12.73	2.33	
Psychological domain	Teleconsultation group	416	13.74	1.16	<0.001
	Usual care group	399	12.35	1.27	
Social relationships domain	Teleconsultation group	416	14.05	1.24	<0.001
	Usual care group	399	12.90	2.39	
Environment domain	Teleconsultation group	416	13.90	1.00	<0.001
	Usual care group	399	12.93	1.20	
Total QOL post-intervention	Teleconsultation group	416	56.19	3.81	<0.001
	Usual care group	399	50.92	5.68	

Table 4 presents the adjusted post-intervention QOL scores across various domains between the teleconsultation and usual care groups. The teleconsultation group demonstrated significantly higher mean scores across all domains: physical health (3.15 vs. 1.38), psychological (1.90 vs. 0.49), social relationships (2.23 vs. 1.09), and environment (1.33 vs. 0.40), with all differences being statistically significant (P < 0.001). Additionally, the total QOL post-intervention score was significantly higher in the teleconsultation group (8.61 vs. 3.36). These results indicate that the teleconsultation group experienced greater improvements in QOL compared to the usual care group.

**Table 4:** Adjusted post-intervention QOL from baseline between groups SE, standard error; QOL, quality of life

Domain	Group	N	Mean	Std. Error	P-value
Physical health domain	Teleconsultation group	416	3.15	0.091	<0.001
	Usual care group	399	1.38	0.92	
Psychological domain	Teleconsultation group	416	1.90	0.059	<0.001
	Usual care group	399	0.493	0.060	
Social relationships domain	Teleconsultation group	416	2.234	0.093	<0.001
	Usual care	399	1.086	0.095	
Environment domain	Teleconsultation group	416	1.33	0.051	<0.001
	Usual care group	399	0.403	0.052	
Total QOL post-intervention	Teleconsultation group	416	8.61	0.22	<0.001
	Usual care group	399	3.36	0.23	

## Discussion

In our study, the mean age of participants in the teleconsultation group was significantly younger, at 68.04 years (SD = 5.986), compared to the usual care group, at 69.70 years (SD = 6.962) (P = 0.0001). This age difference is noteworthy as it suggests that the relatively younger elderly population might be more receptive to or benefit more from teleconsultation services. Comparing our findings with other studies, the study by van den Berg et al. [11] involved a cohort with a mean age of 70 years, closely aligning with our usual care group. Their findings also indicated that younger elderly individuals were more likely to engage with teleconsultation, which could be attributed to better adaptability to technology and potentially fewer comorbidities, allowing for more effective management through teleconsultation.

Our study found no significant difference in gender distribution between the teleconsultation group (36.5% female) and the usual care group (37.1% female) (P=0.781), indicating that gender was not an influential factor in the effectiveness of teleconsultation versus usual care. This aligns with findings from other studies, such as the study by Higginbotham et al. [12], which also found no significant gender differences affecting outcomes in telehealth interventions. However, our study revealed that the teleconsultation group was significantly younger, with a mean age of 68.04 years compared to 69.70 years in the usual care group (P=0.0001). This age difference is relevant, as younger patients may be more adaptable to teleconsultation technologies, as suggested by Manetti et al. [13], who highlighted the role of age in the adoption of digital health platforms.

Socio-demographic details revealed no significant difference in family type (P=0.154). However, the teleconsultation group had a higher proportion of participants with higher education (14.4% vs. 5.8%, P<0.001). This finding aligns with the study by Drosdowsky and Gough [14], which emphasized that higher educational levels may facilitate better understanding and utilization of telehealth services. Additionally, the usual care group had a higher percentage of widowed participants (10.3% vs. 6.8%) and more Muslims (10.1% vs. 5.5%), while the teleconsultation group had a higher proportion of Hindus (93.6% vs. 89.6%); these demographic differences may reflect varying cultural and social dynamics influencing the preference and accessibility of teleconsultation, as indicated in studies by van Esch et al. [15] and Ilić et al. [16], which explored the impact of socio-demographic factors on health outcomes and the adoption of health interventions.

In the present study, a significant difference in mean age was reported. Similarly, the study by Georgeton et al. [17], which included participants with a mean age of 86.1 years - slightly older than our teleconsultation group - found that age did not significantly impact the effectiveness of teleconsultation. However, younger elderly participants showed a slight tendency towards better outcomes, likely due to better general health and technological adaptability. In contrast, the study by Mohd Zambri et al. [18], which focused on a slightly younger population with a mean age of 72.9 years, observed that while teleconsultation was beneficial across all age groups, the impact was more pronounced in the younger segment of the elderly population. This echoes our findings that younger elderly participants might derive more substantial benefits from teleconsultation interventions. The research by Montani et al. [19], which included participants with a mean age of 88 years, also highlighted the significance of age. Their findings suggested that frailty and comorbidities, which tend to increase with age, were critical factors influencing the effectiveness of teleconsultation. Thus, while teleconsultation was effective across various age groups, younger elderly participants, who typically have fewer comorbidities, benefited more. Overall, our study aligns with the existing literature, indicating that younger elderly individuals are more likely to benefit from teleconsultation, potentially due to better overall health, fewer comorbid conditions, and greater ease in adapting to and using telehealth technologies.

In the present study, baseline QOL scores were notably higher in the teleconsultation group across most domains, which aligns with findings from Van Esch et al. [15] and Ilić et al. [16], who emphasized the importance of using validated tools like WHOQOL-BREF for assessing QOL. This higher initial health status in the teleconsultation group may have contributed to their improved post-intervention QOL scores.

Our study observed significant post-intervention improvements in QOL across all domains for the teleconsultation group compared to the usual care group. These results are consistent with several other studies evaluating telehealth interventions. For instance, Baker et al. [20] conducted a multisite RCT with heart failure patients, where the intervention group, receiving intensive education and self-care training, showed substantial gains in self-efficacy and overall QOL. Similarly, Magid et al. [21] reported significant reductions in blood pressure and enhanced health outcomes in patients with hypertension who participated in a multimodal intervention, suggesting that better baseline health can amplify the benefits of telehealth interventions.

Furthermore, Antonicelli et al. [22] found that regular telehealth interactions improved medication adherence and reduced hospital readmissions among heart failure patients, leading to better health outcomes and QOL. Our study supports this, demonstrating that the teleconsultation group achieved significantly better post-intervention QOL scores, likely due to continuous telehealth support.

An adjusted analysis was conducted to account for the higher baseline QOL scores in the teleconsultation group. This adjustment ensured that observed improvements in post-intervention QOL were attributable to the teleconsultation intervention itself rather than pre-existing differences. Our findings reveal that teleconsultation notably enhanced QOL across all domains, including physical health, social relationships, psychological well-being, and environmental factors, in comparison to usual care.

These results align with the work of Esterle and Mathieu-Fritz [23] and Piotrowicz et al. [24], who also reported significant improvements in QOL with telehealth interventions. Our study, in particular, highlights superior patient outcomes in QOL for the teleconsultation group, underscoring the effectiveness of telehealth in enhancing both physical and psychological health. The consistent rise in QOL scores across various studies highlights the effectiveness of telehealth in providing continuous support, education, and monitoring, which are crucial for better management of chronic conditions and overall patient well-being. Our study further supports this, demonstrating that teleconsultation significantly improves health outcomes and QOL for elderly patients. The notable improvements in post-intervention QOL scores across all domains in the teleconsultation group underscore the effectiveness of telehealth in managing chronic conditions and enhancing patient well-being.

Study limitations

This study had several limitations. It relied on participants using ISO-certified home-based devices for self-monitoring key health parameters, and compliance with these protocols was not directly overseen by researchers, potentially introducing variability in data accuracy and reliability. Additionally, only patients who already possessed these instruments at home were included, excluding those without such devices. Furthermore, although allocation concealment was achieved using SNOSEs, blinding of participants and healthcare providers was not possible due to the nature of the intervention. This lack of blinding may have introduced performance or detection bias, as participants aware of their assignment to the intervention group might have altered their behavior, including increased adherence to medical advice.

Study implications

The findings of this study emphasize the potential of teleconsultation as a highly effective method for enhancing the QOL in geriatric patients aged 60 and above. By providing accessible and convenient healthcare services remotely, teleconsultation can effectively address the physical, psychological, and social challenges that elderly patients, particularly those with chronic illnesses, often face. This approach not only reduces the strain on traditional healthcare systems but also improves the overall well-being of older adults, making it a valuable tool in geriatric care.

## Conclusions

This study demonstrates that teleconsultation significantly improves the QOL for elderly patients by offering a convenient, accessible, and effective means of healthcare delivery. The teleconsultation group consistently outperformed the usual care group across all measured domains, highlighting its potential to address the unique challenges faced by the geriatric population, including mobility issues, chronic disease management, and frequent hospital visits. Despite potential limitations such as digital literacy and infrastructure challenges in India, teleconsultation presents a promising solution to alleviate the growing pressure on healthcare systems.
